# Outcomes after ABO incompatible pediatric liver transplantation are comparable to ABO identical/compatible transplant

**DOI:** 10.3389/fped.2023.1092412

**Published:** 2023-05-31

**Authors:** Caroline P. Lemoine, Katherine A. Brandt, Mahima Keswani, Riccardo Superina

**Affiliations:** ^1^ Division of Transplant and Advanced Hepatobiliary Surgery, Northwestern University Feinberg School of Medicine, Chicago, IL, United States; ^2^ Division of Nephrology, Ann & Robert H. Lurie Children’s Hospital of Chicago, Northwestern University Feinberg School of Medicine, Chicago, IL, United States

**Keywords:** pediatric liver transplantation, ABO incompatible liver transplantation, isohemagglutinin titers, plasmapheresis, vascular and biliary complications

## Abstract

**Background:**

ABO incompatible (ABOi) liver transplantation (LT) was initially associated with a higher incidence of vascular, biliary, and rejection complications and a lower survival than ABO compatible (ABOc) LT. Various protocols have been proposed to manage anti-isohemagglutinin antibodies and hyperacute rejection. We present our experience with a simplified protocol using only plasmapheresis.

**Methods:**

A retrospective review of all patients who received an ABOi LT at our institution was performed. Comparisons were made based on era (early: 1997–2008, modern: 2009–2020) and severity of disease (status 1 vs. exception PELD at transplant). A pair-matched comparison was done to patients who received an ABOc LT. *p* < 0.05 was considered significant.

**Results:**

17 patients received 18 ABOi LT (3 retransplants). Median age at transplant was 7.4 months (1.1–28.9). 66.7% patients were listed as status 1. Hepatic artery thrombosis (HAT) occurred in one patient (5.6%), there were 2 cases of portal vein thrombosis (PVT) (11.1%), and 2 biliary strictures (11.1%). Patient and graft survival improved in the ABOi modern era, although not significantly. In the pair-matched comparison, complications (HAT *p* = 0.29; PVT *p* = 0.37; biliary complications *p* = 0.15) and survival rates were similar. Patient and graft survivals were 100% in the non-status 1 ABOi patients compared to 67% (*p* = 0.11) and 58% (*p* = 0.081) respectively for patients who were transplanted as status 1.

**Conclusion:**

ABO incompatible liver transplants in infants with a high PELD score have excellent outcomes. Indications for ABO incompatible transplants should be liberalized to prevent deaths on the waiting list or deterioration of children with high PELD scores.

## 1. Introduction

According to the latest report from the Organ Procurement and Transplantation Network (OPTN), in 2020 the pediatric liver transplant (LT) waitlist mortality decreased to its lowest rate since 2011. However, the rate remains the highest in small children less than 1 year of age with a range of 6.4 deaths per 100 waitlist-years (1). Additionally, small children less than 1 year old continue to constitute the largest group of children waiting for a LT.

ABO incompatible (ABOi) LT is a strategy to help increase the donor pool. Although its utilization has increased in the latest OPTN report, it remains rarely utilized (5.6%). Reports from the early experience with ABOi grafts in pediatric and adult LT suggested a greater incidence of antibody-mediated (“hyperacute”) rejection, vascular thrombosis, and biliary complications (2–4). Circulating preformed antibodies to the donor blood group have been shown to bind to the graft endothelium, leading to complement activation (3).

Patient and graft survival were also noted to be lower than with ABO identical or compatible (ABOc) grafts (5, 6). However, recently published studies showed improved outcomes after ABOi (7, 8). The age of the recipient was shown to be an important predictive factor of outcomes, where age greater than 1 year at the time of ABOi LT was associated with an increased morbidity and mortality (9, 10).

Elevated anti-ABO titers were also found to be predictive of outcomes (9). Various strategies have been reported to help prevent the binding of preformed iso-hemagglutinins to the graft vasculature, such as splenectomy, plasmapheresis (PLEX), and Rituximab (6, 9, 11, 12). Management protocols based on pre-LT anti-ABO titers have also been developed (11).

We have been performing ABOi pediatric LT since the early years of our program, utilizing a simplified protocol solely involving PLEX after LT. Here we present our institutional ABOi management protocol as well as results with ABOi pediatric LT in the early and modern eras of our program. We hypothesize that the results are comparable to those after ABOc LT.

## 2. Material and methods

### 2.1. Study population and data collection

A retrospective review of our institutional prospectively collected database of patients who received a LT at our institution was performed after IRB approval was obtained (IRB #2013-15357).

The following data was collected: sex, indication for transplant (diagnosis), age at listing and age at transplant, height and weight at transplant, status at transplant, calculated and exception Pediatric End-stage Liver Disease (PELD) score at transplant, ABO of recipient. Transplant related data included type of graft, ABO of donor, cold ischemia time, surgery duration, intraoperative and postoperative (<24 and <72 h after LT) packed red blood cell (pRBC) transfusions, length of hospital stay. Complications that were recorded included hepatic artery thrombosis (HAT), portal vein thrombosis (PVT), bile leak, biliary stricture, biopsy proven rejection episodes, and post-transplant lymphoproliferative disease (PLTD). Vascular and biliary complications were categorized as early (≤30 days from transplant) or late (>30 days from transplant). Graft and patient status at last follow-up were noted. Cause of graft failure or patient death were recorded. Iso-hemagglutinin titers were collected pre-transplant as well as at various points post-transplant (detailed in the next section). Of note, A2 donors donating to O recipients was not considered an ABOi LT as the results of those LT differ from “true” ABOi with excellent outcomes and survival (13, 14).

A retrospective analysis of the United Network for Organ Sharing (UNOS) data of all pediatric patients (age <18 years at transplant) who received a liver transplant alone between January 1st, 1997, and November 30th, 2020 was performed to determine the national yearly proportion of ABOi performed throughout the United States.

### 2.2. ABOi LCH protocol

According to United Network for Organ Sharing (UNOS) regulations (15) since 2006, when a patient aged 12 months or less reaches a natural PELD score of at least 30 due to severity of disease and in the absence of suitable ABO identical or ABO compatible (ABOc) grafts (including technical variants and living donor grafts) and after multidisciplinary agreement, the patient is listed across all blood types including ABO incompatible (ABOi) (before 2006, a minimum PELD score of 25 was acceptable for ABOi listing). Baseline anti-iso-hemagglutinin titers (anti-A and anti-B) are measured in all recipients regardless of the recipient and the donor blood groups. When an ABOi graft is accepted, a temporary central venous dialysis catheter is placed by the interventional radiology team in preparation for post-transplant PLEX. No other pre-transplant intervention is performed regardless of the baseline iso-hemagglutinin titers. Rituximab is not administered as part of our ABOi institutional management.

Induction immunosuppression consists of methylprednisolone (10 mg/kg) given intravenously once intraoperatively (before 2006, the induction immunosuppression consisted of a combination of azathioprine, basiliximab, and methylprednisolone). In patients with hepatorenal syndrome or acute or chronic renal insufficiency, basiliximab is also administered postoperatively on arrival in the pediatric intensive care unit (10 mg/dose for patients <35 kg and 20 mg/dose for patients ≥35 kg). A second dose (same dosage) is given on the fourth postoperative day. Intraoperatively and postoperatively, packed red blood cell transfusions (pRBC) consist of recipient ABO blood group while fresh frozen plasma (FFP) and platelet transfusions are from the donor ABO blood group due to antigens contained in FFP. Donor specific antibodies (DSA) are not routinely measured prior to LT at our institution.

Our institutional fluid and anticoagulation post-transplant management has been presented in a previous publication (16). Importantly, postoperative Jackson-Pratt drain output is replaced with FFP for the first 5 days post LT. Also, split and reduced size grafts receive prostaglandin E1 for 5 days to minimize ischemia reperfusion injury. Postoperative immunosuppression is not modified based on ABO compatibility. All patients receive an intravenous methylprednisolone taper starting at 1 mg/kg/dose twice a day until reaching 0.3 mg/kg/dose once a day. Steroids are continued at the same dose until 3 months post-transplant after which they are progressively weaned and ultimately stopped between 5 and 8 months after surgery. Maintenance immunosuppression consists of mycophenolate mofetil (10 mg/kg/dose, maximum dose 1,000 mg/dose) and tacrolimus (0.1 mg/kg/dose, maximum dose 5 mg/dose, goal level 10–12 ng/ml for the first 3 months post-transplant). Both are started on postoperative day 0 or 1. Prior to 2006, the maintenance immunosuppression relied on cyclosporine. Surveillance liver biopsy is performed every 5 years after transplantation.

Anti-isohemagglutinin titers are generally measured on postoperative days 1, 3, 5, 7, and 9. Low antibody titers are considered at anything below 1:8 dilution. Five PLEX sessions are performed on postoperative days 2, 4, 6, 8, 10 using donor FFP. Patients undergo 5 PLEX sessions regardless of the variation in iso-hemagglutinin titers (even if titers are not detectable before completion of the fifth sessions). For small patients (<5 kg) who are too small either to tolerate PLEX due to volume shifts or too small for a dialysis central venous catheter, double-volume exchange transfusions is performed in lieu of PLEX.

### 2.3. Pair matching with ABO compatible transplants

In order to assess the outcomes of ABOi LT in comparison to expected results, a pair-matching analysis was performed with ABOc transplants performed at our institution. Pair-matching was performed based on the following criteria: era of transplant ( ± 2 years from ABOi transplant date), age at transplant ( ± 2 years from age at transplant for patients who received an ABOi LT), severity of disease at transplant (matched to the closet severity level: for example status 1A to 1A, status 1B to 1B; when this was not possible, matching was made to the highest exception PELD score; if no matches were identified based on the previously presented options, the match was made to the closest calculated PELD at the time of transplant), and categorized indication for transplant. Categorized indications included biliary atresia (BA), cholestasis, metabolic disease, fulminant liver failure, cirrhosis, retransplantation, and other. When multiple possible matches were found for a same ABOi transplant, the match closest in age at transplant was chosen for comparison.

In the United States, patients are listed for LT according to the PELD or Median End-Stage Liver Disease (MELD) scoring system (based on age younger or older than 12 years of age at the time of transplant). It reflects the 90-day patient mortality risk (15). The PELD score is based on bilirubin, INR, and albumin values, and patient growth. In certain instances, the patient can be listed using a status 1A or status 1B. To qualify as a status 1A, the patient must be suffering from acute liver failure or acutely decompensated Wilson's disease. Also, in patients who underwent a previous transplant, patients who are suffering from HAT or primary non-function also qualify for 1A status. Patients with organic acidemia or urea cycle defect diseases or hepatoblastoma can be listed as status 1B. Patients with chronic liver disease who have a natural PELD score greater than 25 and require mechanical ventilation, continuous renal replacement therapy, or those who suffer from significant gastrointestinal bleeding or hepatic encephalopathy also qualify for status 1B listing.

### 2.4. Statistical analysis

Continuous variables were compared using the independent *t*-test while categorical variables were evaluated using the *χ*^2^ test. Categorical variables with more than 2 levels were compared using multinomial logistic regression. Kaplan–Meier curves were generated to compare patient and graft survival.

Comparisons were made based on the era of our transplant program in which the transplant was performed (early era: 1997–2008 vs. modern era: 2009–2020), ABO compatibility (ABOc vs. ABOi), and on severity of disease at transplant (patients transplanted as status 1, 1A, or 1B vs. those transplanted with a PELD score). Statistical analysis was performed using the IBM SPSS Statistics program (version 25.0.0.0). A *p* value <0.05 was considered statistically significant.

## 3. Results

### 3.1. ABO incompatible liver transplants

Between August 1st, 1997, and December 31st, 2020, a total of 18 ABOi LT were performed in 17 patients. All patients and transplants were included including patients receiving a retransplantation. There was no multivisceral or multiorgan transplantation. All ABOi grafts were obtained from deceased donors.

**Figure 1:**
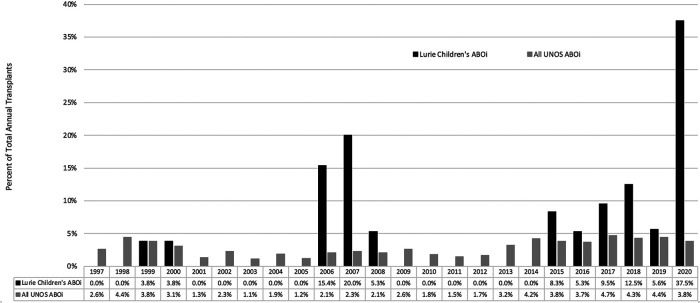
Proportion of ABO incompatible liver transplants by year, LCH and UNOS data.

The overall proportion of ABOi LT at our institution was 4.1%. Figure 1 presents the proportion of ABOi transplants that were performed on a yearly basis both at our institution and nationally (based on UNOS data).

The median age at transplant was 224 days (range 31 days-28 months) and the median weight was 7.1 kg (range 3–12.8 kg). Three patients were older than 12 months old at the time of transplant and all transplanted in the early era: the first patient who received an ABOi was 22-month-old in 1999, while the 2 others were 20 and 28 months old (transplanted in 2006 and 2007). Since then, all patients have been less than 12 months old at the time of transplant.

The indications for LT were BA (*n* = 7, 39%), fulminant liver failure (*n* = 3, 17%), metabolic disease (*n* = 2, 11%), gestational alloimmune liver disease (GALD) (*n* = 2, 11%), and cryptogenic cirrhosis (*n* = 1, 6%). Three ABOi were re-transplants. In 12 cases, patients were transplanted as Status 1 (2 patients were Status 1 before the PELD system; 7 patients were status 1A, and 3 patients were status 1B). The other 5 patients were transplanted with a median exception PELD of 37 (range 27–48).

A whole liver transplant was performed in 5 cases, split transplant in 7 cases, and a reduced size graft in 6 cases. The portal vein was anastomosed in an end-to-end fashion in most cases (14/18), and in 4 cases, a donor iliac vein interposition graft was used. The celiac axis of the donor was most often anastomosed to the recipient's aorta (9/18). A donor iliac arterial interposition graft was used in 2 cases. The biliary anastomosis was a Roux-en-Y hepaticojejunostomy in 17 cases. There was only one duct-to-duct anastomosis. Most ABOi LT were A donor to O recipient (*n* = 8) and AB donor to A recipient (*n* = 5). Other transplants included B donor to O recipient (*n* = 2), AB donor to O recipient (*n* = 1), A donor to B recipient (*n* = 1), and AB donor to B recipient (*n* = 1).

Early HAT occurred in one patient (5.6%) in the ABOi cohort. It occurred on postoperative day 1, could not be revascularized, and required a re-transplantation. There were 2 late HAT, one at 5 years post-transplant and one at 7 months after LT. Both grafts are functional at last follow-up (12 years and 2.9 years), as the intrahepatic arterial system was revascularized through the Roux limb arterial supply.

The incidence of early portal vein thrombosis (PVT) was 5.6% (1/18). It was successfully thrombectomized on postoperative day 5. There was one late PVT occurring 5 years post-transplant in a different patient, the same patient who suffered from a late HAT. The patient has no sign or symptom of portal hypertension.

There were 5 biliary complications: three early biliary leaks (16.7%), 2 requiring an anastomotic revision, while the third one was a cut surface leak. There were 2 biliary anastomotic strictures (11.1%), both requiring a percutaneous transhepatic cholangiography with balloon dilatation and stent placement. One of those 2 grafts was ultimately lost to chronic rejection and biliary complications and needed a retransplantation. This patient first suffered from an anastomotic bile leak requiring a surgical revision, which was complicated by an anastomotic stricture. There was no case of generalized ischemic cholangiopathy.

Six patients suffered from acute rejection (33.3%). One child suffered from chronic rejection after her immunosuppression had to be withdrawn due to a severe adenovirus systemic infection. She was listed for retransplantation. This was not an episode of antibody mediated rejection (the C4d staining was negative on biopsy).

The overall patient survival was 76.5% (13/17). Both patients who suffered from GALD died during their transplant admission (one from sepsis and the other one from hemorrhagic shock from a dislodged neck hemodialysis line): both patients had a functioning graft at the time of death. They were 4 and 5 weeks old and weighed 3.0 and 3.3kg respectively. Of note, the hemodialysis line was not used for PLEX but for dialysis due to acute kidney injury postoperatively. A third patient also died during the transplant admission: he suffered from liver failure from mitochondrial disease. He developed cardiac mitochondrial disease complications and suffered from a fatal arrythmia. He was not a heart transplant candidate. His graft was functioning at the time of death. The last patient died during re-transplantation for chronic rejection 9 months after the initial transplant from cardiac arrythmias.

Only one patient who was older than 12 months at the time of transplant is still followed at our institution. She was transplanted when she was 28 months old and is still alive with good graft function 12.5 years after LT. The patient who was 20 months old died during retransplantation for chronic rejection. The patient who was 22 months old transferred care to another institution but was alive at the time of transfer 5 years posttransplant.

The overall graft survival was 72.2% (13/18). Three grafts were lost to patient death and one graft was lost to chronic rejection. Lastly, one graft was lost to HAT leading to retransplantation.

### 3.2. Comparison based on compatibility

**Figure 2:**
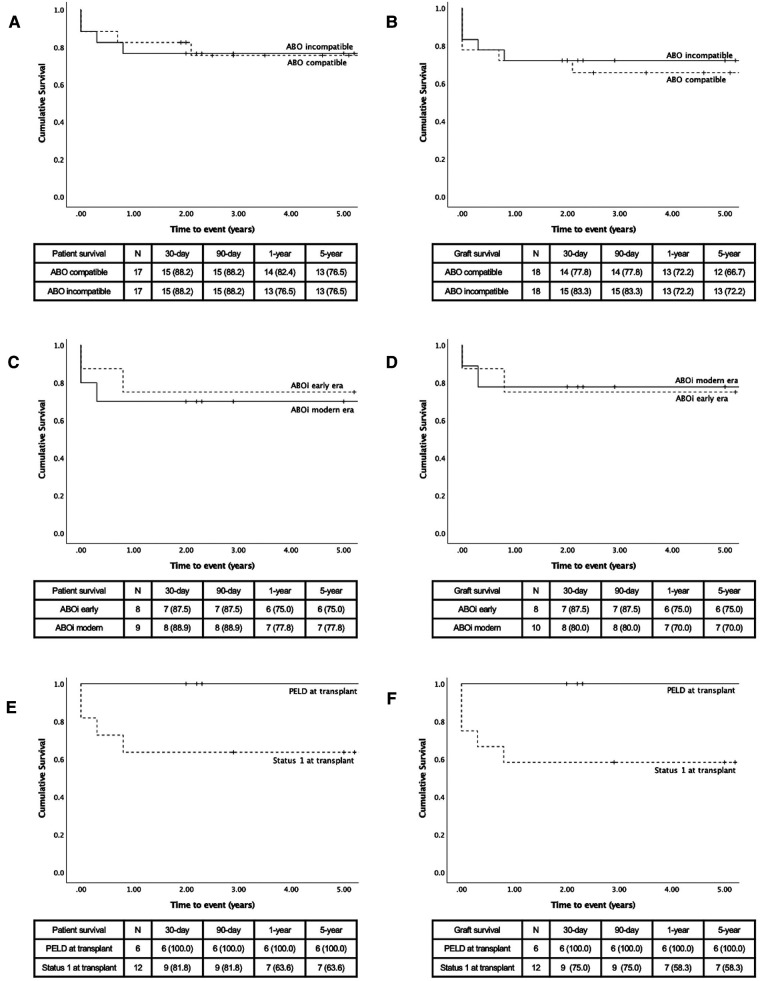
Kaplan–Meier survival curves, comparison made by compatibility (**A**) patient survival and (**B**) graft survival; by era (**C**) patient survival (**D**) and graft survival; by status at transplant (**E**) patient survival and (**F**) graft survival.

The comparisons of ABOc and ABOi LT are presented in Table 1. As expected by the pair-matching criteria, there was no difference between groups based on age, severity of disease, and indication for LT. There was no significant difference between the transplant variables or post-LT complications, although the rate of early HAT (16.7% vs. 5.6%, *p* = 0.29), re-transplant for HAT (11.1% vs. 5.6%, *p* = 0.55), and early PVT (16.7% vs. 5.6%, *p* = 0.29) all occurred less frequently in the ABOi group. There was no difference in patient survival between groups (Figure 2A). The graft survival was non-significantly higher in the ABOi group (Figure 2B).

**Table 1:** Patient characteristics and outcomes, ABO incompatible vs. ABO compatible transplants. Results are presented either as mean ± sd (continuous) or *n* (%) (categorical).

	Comparison based on compatibility		
	ABO compatible	ABO incompatible	*p* value
	*n* = 17 patients	*n* = 17 patients	
	*n* = 18 grafts	*n* = 18 grafts	
Sex (female)	11 (64.7)	8 (47.1)	0.3
**Diagnosis**			
Biliary atresia	7 (38.9)	7 (38.9)	0.44
Acute liver failure	4 (22.2)	3 (16.8)	
Cholestasis	1 (5.6)	–	
Cirrhosis	–	1 (5.6)	
Metabolic	3 (16.7)	2 (11.1)	
Other	–	2 (11.1)	
Retransplant	3 (16.7)	3 (16.8)	
Waitlist duration (days)	73.7 ± 126.4	51.5 ± 99.9	0.56
Age at transplant (days)	301.4 ± 172.2	264.0 ± 211.5	0.57
Weight at transplant (kg)	7.7 ± 2.7	7.5 ± 2.8	0.78
**Status 1**			
Status 1	2 (11.1)	2 (11.1)	0.9
Status 1A	6 (33.3)	7 (38.9)	
Status 1B	4 (22.2)	3 (16.8)	
PELD exception at transplant	36 ± 4	37 ± 7	0.8
PELD calculated at transplant	23 ± 18	29 ± 15	0.34
**Type of graft**			
Whole	3 (16.7)	5 (27.8)	0.55
Reduced	9 (50.0)	6 (33.3)	
Split	6 (33.3)	7 (38.9)	
Cold ischemia time (h)	9.4 ± 1.9	8.6 ± 1.5	0.21
Operative duration (min)	403.7 ± 103.8	415.8 ± 115.7	0.74
Intraoperative pRBC transfusions (cc/kg)	189.0 ± 206.0	274.0 ± 278.3	0.31
≤24 h pRBC transfusions (cc/kg)	28.2 ± 41.0	28.3 ± 32.1	0.99
≤72 h pRBC transfusions (cc/kg)	376.5 ± 550.0	342.3 ± 391.6	0.83
Hospitalization length of stay (days)	63.2 ± 69.1	57.4 ± 30.0	0.78
**Complications**			
Early HAT (<30 days)	3 (16.7)	1 (5.6)	0.29
Late HAT (≥ 30 days)	0	2 (10.5)	0.15
Retransplantation for HAT	2 (11.1)	1 (5.6)	0.55
Early PVT (< 30 days)	3 (16.7)	1 (5.6)	0.29
Late PVT (≥ 30 days)	1 (5.6)	1 (5.6)	–
Early bile leak (≤ 30 days)	4 (22.2)	3 (16.7)	0.67
Biliary stricture (> 30 days)	1 (5.6)	2 (11.1)	0.55
Rejection	6 (33.3)	6 (33.3)	–
PTLD	1 (5.6)	2 (11.1)	0.55
Follow-up (years)	3.2 ± 4.9	5.3 ± 5.3	0.82

Legend: ABOc, ABO compatible; ABOi, ABO incompatible; HAT, Hepatic artery thrombosis; PELD, Pediatric End-stage Liver Disease; pRBC, Packed red blood cells; PTLD, Post transplant lymphoproliferative disease; PVT, Portal vein thrombosis.

### 3.3. Comparison based on era of transplant

Eight transplants were performed in the early era and 10 in the modern era (Table 2). There was no difference between the two groups in pre-transplant variables, but there was a trend to a younger age at transplant in the modern era (183.0 ± 74.8 days vs. early era 365.3 ± 283.5 days, *p* = 0.067). The groups were also similar when comparing transplant related data. However, the length of the transplant surgery was statistically shorter in the modern era (362.6 ± 87.1 vs. 482.3 ± 116.9 min, *p* = 0.024). There was no difference in postoperative complications. Although non-significant, the rate of early bile leak (25% vs. 10%, *p* = 0.4), rejection (50% vs. 20%, *p* = 0.18) and PTLD (25% vs. 0%, *p* = 0.94) were all less frequent in the modern era.

**Table 2:** Patient characteristics and outcomes after ABO incompatible liver transplants: comparison based on era and status 1 vs. PELD at transplant. Results are presented either as mean ± sd (continuous) or *n* (%) (categorical). Statistically significant results are highlighted in bold font.

	Comparison based on era			Comparison based on status at transplant		
	ABOi early era	ABOi modern era	*p* value	ABOi PELD	ABOi Status 1	*p* value
	*n* = 8 patients	*n* = 9 patients		*n* = 6 patients	*n* = 11 patients	
	*n* = 8 grafts	*n* = 10 grafts		*n* = 6 grafts	*n* = 12 grafts	
Sex (female)	5 (62.5)	3 (33.3)	0.23	3 (50.0)	5 (45.5)	0.86
**Diagnosis**						
Biliary atresia	4 (50.0)	3 (30.0)	0.45	**6 (100.0)**	**1** (**8.3)**	0.004*
Acute liver failure	2 (25.0)	1 (10.0)		**–**	**3** (**25.0)**	
Cholestasis	–	–		**–**	**–**	
Cirrhosis	–	1 (10.0)		**–**	**1** (**8.3)**	
Metabolic	–	2 (20.0)		**–**	**2** (**16.7)**	
Other	1 (12.5)	1 (10.0)		**–**	**2** (**16.7)**	
Retransplant	1 (12.5)	2 (20.0)		**–**	**3** (**25.0)**	
Waitlist duration (days)	87.1 ± 144.0	23.0 ± 26.0	0.18	**119.3 ± 156.2**	**17.6 ± 23.6**	0.037*
Age at transplant (days)	365.3 ± 283.5	183.0 ± 74.8	0.067	297.3 ± 134.9	247.3 ± 244.9	0.65
Weight at transplant (kg)	8.2 ± 3.5	6.9 ± 2.1	0.38	7.7 ± 1.7	7.4 ± 3.3	0.8
**Status 1**						
Status 1	2 (25.0)	–	0.13	–	2 (16.7)	—
Status 1A	2 (25.0)	5 (50.0)		–	7 (58.3)	
Status 1B	1 (12.5)	2 (20.0)		–	3 (25.0)	
PELD exception at transplant	38 ± 11	36 ± 2	0.73	37 ± 7	–	–
PELD calculated at transplant	37 ± 10	24 ± 16	0.09	31 ± 10	28 ± 19	0.66
**Type of graft**						
Whole	2 (25.0)	3 (30.0)	0.37	**2 (33.3)**	**3** (**25.0)**	0.036*
Reduced	4 (50.0)	2 (20.0)		**–**	**6** (**50.0)**	
Split	2 (25.0)	5 (50.0)		**4 (66.7)**	**3** (**25.0)**	
Cold ischemia time (h)	8.3 ± 1.5	8.9 ± 1.5	0.44	8.9 ± 1.8	8.5 ± 1.4	0.58
Operative duration (min)	**482.3 ± 116.9**	**362.6 ± 87.1**	0.024*	424.0 ± 60.4	411.7 ± 137.7	0.84
Intraoperative pRBC transfusions (cc/kg)	205.7 ± 290.7	139.7 ± 91.2	0.27	173.6 ± 73.5	196.7 ± 250.8	0.83
≤24 h pRBC transfusions (cc/kg)	22.6 ± 25.5	32.1 ± 50.2	0.65	30.0 ± 27.1	27.2 ± 48.2	0.9
≤72 h pRBC transfusions (cc/kg)	232.1 ± 252.3	477.6 ± 683.9	0.38	340.5 ± 295.8	396.2 ± 662.6	0.85
Hospitalization length of stay (days)	65.7 ± 28.2	49.1 ± 31.1	0.32	50.8 ± 37.4	62.4 ± 24.2	0.5
**Complications**						
Early HAT (<30 days)	0	1 (10.0)	0.36	0	4 (33.3)	0.11
Late HAT (≥ 30 days)	1 (12.5)	1 (10.0)	0.87	0	3 (25.0)	0.18
Retransplantation for HAT	0	1 (10.0)	0.36	0	1 (8.3)	0.47
Early PVT (< 30 days)	0	1 (10.0)	0.36	0	1 (8.3)	0.47
Late PVT (≥ 30 days)	1 (12.5)	0	0.25	0	1 (8.3)	0.47
Early bile leak (≤ 30 days)	2 (25.0)	1 (10.0)	0.4	0	3 (25.0)	0.18
Biliary stricture (> 30 days)	1 (12.5)	1 (10.0)	0.87	0	2 (16.7)	0.29
Rejection	4 (50.0)	2 (20.0)	0.18	2 (33.3)	4 (33.3)	–
PTLD	2 (25.0)	0	0.094	1 (16.7)	1 (8.3)	0.47
Follow-up (years)	**8.5 ± 6.1**	**2.4 ± 2.2**	0.013*	7.4 ± 6.8	4.1 ± 4.2	0.23

Legend: (*) denotes a statistically significant result; ABOi, ABO incompatible; HAT, Hepatic artery thrombosis; PELD, Pediatric End-stage Liver Disease; pRBC, Packed red blood cells; PTLD, Post transplant lymphoproliferative disease; PVT, Portal vein thrombosis.

Patient and graft survivals are presented in Figures 2C,D. The patient survival was non-statistically superior in the modern era. The cause of patients' deaths was presented earlier. All patients who died in the modern era had a functional liver graft at the time of death. Graft survival was non-significantly higher in the early era. In the modern era, graft losses were cause by 2 patient deaths (previously described) and by one early HAT. Graft losses in the early era were both related to patient mortality.

### 3.4. Comparison based on severity of disease

Eleven patients received an ABOi graft for status 1, 1A, or 1B. All 6 patients who received an ABOi LT with a PELD score (non-status 1) were transplanted for BA. This was significantly different from the patients transplanted as status 1 where only one patient was transplanted for BA (*p* = 0.004) (Table 2). The waitlist duration was shorter in the status 1 group (17.6 ± 23.6 days vs. 119.3 ± 156.2 days, *p* = 0.037). While there was no statistical difference in the occurrence of postoperative complications, there were no vascular or biliary complications in the patients transplanted with a PELD score.

The 5-year patient and graft survivals were 100% in patients transplanted with a PELD score (Figures 2E,F). Although the patient survival was 63.6% at one-year in the status 1 group, the difference was not statistically significant (*p* = 0.11). There was a trend to a lower graft survival in the status 1 group (58.3%, *p* = 0.081).

### 3.5. Plasmapheresis: Variation of iso-hemagglutinin titers post-transplant

All patients were able to complete the 5 planned sessions of PLEX stipulated in our protocol except for the 2 smaller patients who suffered from GALD. They received double-volume exchange transfusions every 48h. There were no complications from PLEX.

**Figure 3:**
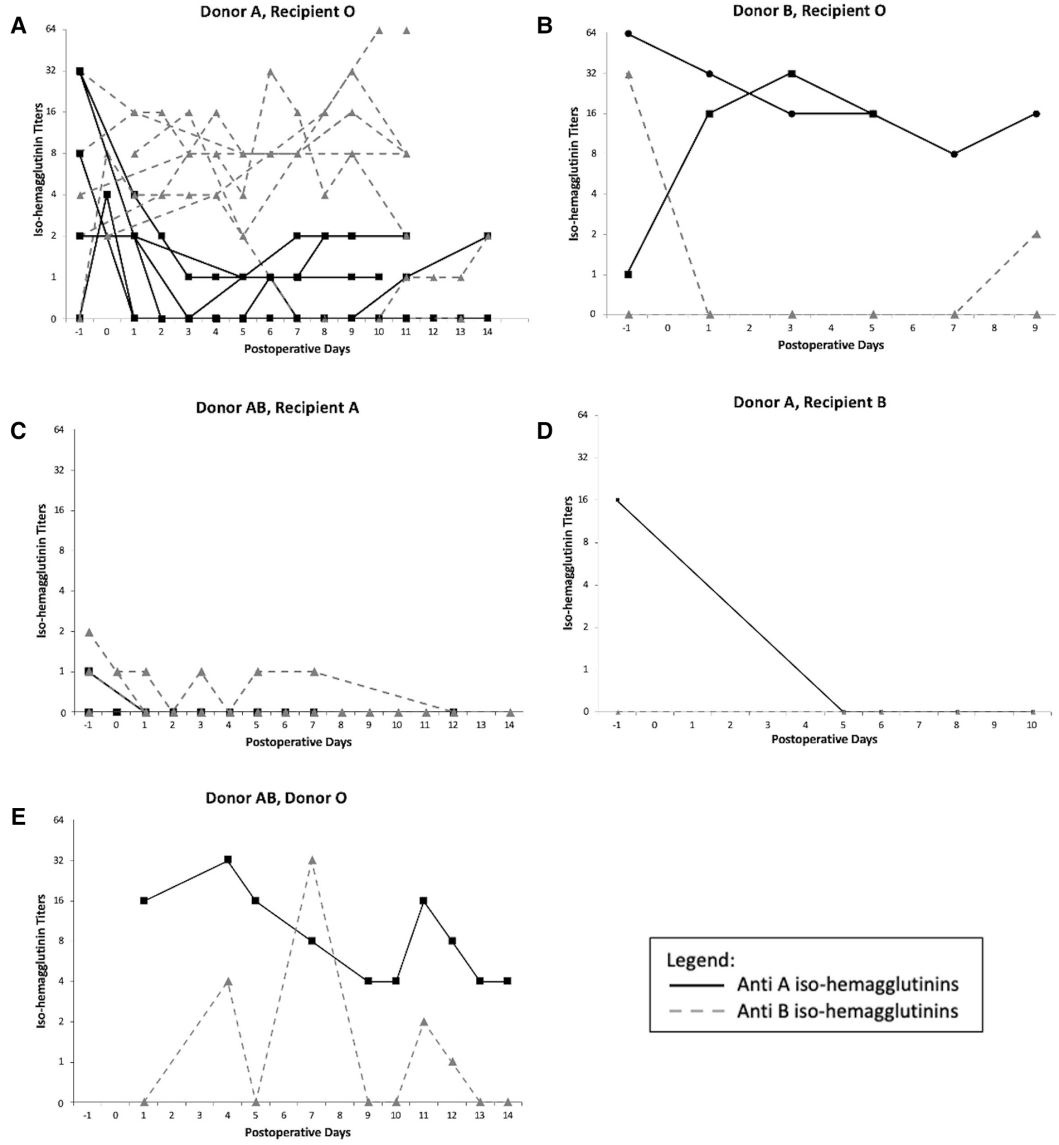
Iso-hemagglutinins titers variation after ABO incompatible liver transplant: (**A**) Donor A, Recipient O, (**B**) Donor B, Recipient O, (**C**) Donor AB, Recipient A, (**D**) Donor A, Recipient B, (**E**) Donor AB, Recipient O. −1 represents the titers measured pre-LT.

The variation of iso-hemagglutinin titers before and after LT is presented in Figure 3. In 5 blood group O recipients who received a donor A graft, there were variable pre-transplant anti-A titers, between 1:2 and 1:32. They were undetectable in one patient and were missing in 2 other patients. Post LT, all the anti-donor-specific titers decreased to no higher than 1:2 for the duration of the plasmapheresis. In contrast, the anti-B titers remained relatively high in 5 patients, but were undetectable in one patient.

The same pattern was observed in the donor B in recipient O ABOi LT: the anti-A titers were high in one patient and low in the second before LT (1:1 and 1:64) and remained high (between 1:8 and 1:32) for the duration of the PLEX treatment in both patients, while the anti-B titers were high in one patient, undetectable in the second one, and both remained low for the duration of the treatment.

For the donor AB in recipients A ABOi LT, the anti-B titers were low in one patient and undetectable in the other, and they remained either lower or undetectable during PLEX. The anti-A titers were mildly detectable pre-transplant and became undetectable during the duration of the treatment. A similar pattern was observed in the donor A into recipient B LT.

For the donor AB into recipient O ABOi LT, AB plasma given to the patient post-transplant should not have contained either anti-A or anti-B iso-hemagglutinins. This was the first ABOi LT of our program done 25 years ago. While we cannot explain the variation of the anti-iso-hemagglutinins during the PLEX treatment or after it was completed, the patient did not suffer from any hyperacute rejection or post-LT rejection despite them being detectable.

There was only one donor AB in recipient B ABOi LT. The graft was lost in the first 24h after transplant and the patient was retransplanted as a status 1A. No titers were therefore measured after transplant.

## 4. Discussion

The historical experience with ABOi LT has been associated with decreased patient and graft survival. More recent publications describe outcomes comparable to ABOc graft, including similar rates of vascular and biliary complications, rejection, and patient and graft survival (7, 12). Our institutional experience with pediatric ABOi LT aligns with those recent reports. Early HAT leading to re-transplantation was low at 5.6%. Late biliary strictures (11.1%) and chronic rejection (5.6%) were uniformly low. All complications could be explained by non-immune mechanisms and were comparable to these observed in the ABOc LT group. Importantly, there was no occurrence of hyperacute AMR. These improved results after ABOi reflect improvements in surgical technique, perioperative medical management (including immunosuppression), and the utilization of our institutional ABOi management protocol that relies solely on PLEX.

Many strategies have been reported to help prevent the deleterious effects of circulating preformed antibodies in ABOi LT. Splenectomy is now rarely used given other medical strategies than can be utilized (17, 18). Rituximab is increasingly utilized as pre-treatment before ABOi LDLT, thus decreasing the number of circulating antibody forming B lymphocytes (19). Known complications include leukopenia, renal dysfunction, pulmonary edema, hypotension, and allergic reactions (20). It may also predispose patients to infectious complications. Given the good outcomes with ABOi LT in children less than 1 year given the immaturity of their immune system and as they have less complement system activation than adults (21, 22), Rituximab is not necessary in this specific population.

PLEX has been shown to effectively reduce anti-donor iso-hemagglutinin levels (23). Although a generally safe technique, catheter related sepsis and dislodgment remain potential concerns. At our program, we follow institutional CLABSI prevention protocols and because the catheters are kept in place only for a short duration (10 days), the risk of infection is low, and no patient suffered from CLABSI in our experience. There was no dislodgment of a PLEX specific line, but one patient with GALD (3.5 kg) who developed acute kidney injury and required continuous renal replacement therapy died from hemorrhagic shock from a dislodged dialysis catheter, the same type of catheter used for PLEX. The patient had lost a significant amount of body wall edema with fluid removal which made the line more mobile in the soft tissues. Ensuring catheters are well secured multiple times a day and taking special precautions when the patient is moved are paramount to prevent catheter dislodgment and potentially catastrophic complications.

In some studies, direct portal vein or hepatic artery infusions have been utilized to deliver agents directly to the vascular endothelium of the ABOi graft, a technique believed to prevent AMR more successfully than systemic delivery (24, 25). While this technique is now performed less frequently, one of the reportedly infused substances was prostaglandin E1 (PGE1). PGE1 improves microcirculation through vasodilatation and by preventing platelet aggregation (19). Although PGE1 is not part of our ABOi protocol, we administer it in all split and reduced size grafts (16). Since most ABOi LT in our experience have been split or reduced size grafts (13/18, 72.2%), the vasodilatory effect of PGE1 may have been beneficial and contributing to the good early ABOi graft outcomes.

A recent multicenter European collaborative report described the outcomes after ABOi and presented the various perioperative medical management strategies for ABOi LT (8). There was a high variability in practice, with no one strategy superior to the others. Ultimately, institutions should adopt a protocol that leads to good patient and graft outcomes with minimal patient morbidity and maximizes simplicity, regardless of if this protocol differs from others.

In our experience, all ABOi grafts were obtained from deceased donors. However, in countries where access to deceased donors is less readily available such as Japan and Saudi Arabia, living donor ABOi have been successfully performed (26, 27). This constitutes another way of increasing the donor pool for small children <1 year old on the LT waiting list.

Our study showed a decrease in donor-specific iso-hemagglutinin after ABOi LT, while third party antibodies continued to be detected. The latter can be explained by the administration of donor specific FFP which contains non-donor directed circulating iso-hemagglutinins. In addition, we have unpublished evidence that the titers previously reported as low even in the absence of plasmapheresis may be due to the donor specific anti iso-hemagglutinins being adsorbed on to the donor liver endothelium. Liver biopsies stained for anti-endothelial antibodies to the vascular endothelium may be positive. This negates the reassuring absence of a detectable DSA in the recipient serum reported by others as evidence that antibody monitoring may serve as a guide to the need for PLEX. Similar to how the liver exerts a protective effect on kidney transplants from the same donor with a positive cross match in regard to anti HLA antibodies by absorbing the antibodies, the same effect may be at work in the case of anti-hemagglutinin antibodies. However, to prove this latter hypothesis would require repeated liver biopsies and specific staining for DSA, which we have not performed in the setting of this study.

There is evidence that over the long term of several weeks or months after LT the graft reduces the expression of donor ABO blood group on the endothelium and assumes the phenotype of the recipient blood group (28). Thus, the long term consequences of ABOi LT are mitigated by the loss of cells, at least in part, that express or display incompatible ABO blood groups.

In our protocol, all patients receive post LT PLEX regardless of their pre-LT donor-specific titers. PLEX is efficient at reducing anti-A and anti-B titers. A recently published study recommended an aggressive management for patients with titers >1:16 (Rituximab, IVIG, PLEX) while not administering any treatment to patients with lower titers (11). The 1:16 anti-donor antibody titer was reported in the Japanese registry as being associated with a higher incidence of AMR after ABOi adult LDLT, therefore recommending pre-LT PLEX when titers were above 1:16 (29). However, although higher titers have been associated with increased morbidity and mortality (9), others have questioned their prognostic utility.

Most children being listed for an ABOi LT are acutely sick and are coagulopathic before LT, and often receive blood products including FFP that will contain iso-hemagglutinins against non-self-antigens. This passive administration of potential anti-donor antibodies contributes to the higher titers observed in some of our patients' pre-LT and is not due to inherent antibody production by the sick infant.

Our protocol changes the post LT fluid replacement to donor specific FFP that then contains no antibodies that could potentially damage the graft, and explains the higher titers of third party, non-donor directed iso-hemagglutinin antibodies.

Our results support the favorable outcomes observed after ABOi LT in children less than one year of age. Infants less than one year of age have an immature complement system (21) and do not produce iso-hemagglutinins antibodies early in life. Maternal iso-hemagglutinins traverse the placenta from the 18th week of gestation, and they inhibit the development of fetal and neonatal iso-hemagglutinins after birth. Maternal antibodies disappear in the first 2 weeks of life, and by 8–12 weeks, the newborn starts producing antibodies, which leads to anti-A and anti-B titers rising in the first year of life (30). During the first 2 years of life, the titers double and reach full adult levels by age 5–10 (22).

While the outcomes after ABOc and ABOi LT were similar in our series, there was a significantly lower patient and graft survival for ABOi LT in children who were transplanted as status 1 compared to those transplanted with a PELD score. It has previously been shown that worst outcomes observed after ABOi were attributable to the patient's medical urgency at the time of LT rather than to the incompatibility (2, 31). Under current UNOS regulations, a patient's natural PELD must be at least 30 to qualify for ABOi listing. Given the good results observed in ABOi LT in children transplanted under more elective circumstances, we recommend that UNOS regulations should be modified to reduce the minimal natural PELD score making a child less than 12 months old eligible for ABOi. By transplanting patients with a lower PELD score, we believe the outcomes after ABOi would be improved while helping reduce the waitlist mortality.

Some groups have reported excellent patient and graft survival after ABOi, as high as 100% (7, 11, 12). However, they failed to specify what blood group incompatibility they included. For example, in the UNOS database, A2 grafts transplanted in O recipients is frequently mislabeled as an ABOi although it is now well accepted that this ABO combination is equivalent to an ABOc transplant. It is possible their excellent results are in part the result of this favorable transplant combination. We did not consider A2 to O transplants as ABOi LT.

The authors recognize limitations to their study. It is a single center retrospective study with a small number of patients. However, most reports on pediatric ABOi LT are single-center study with a number of patients similar to ours. Also, the study spans over 25 years, and while the ABOi management protocol did not change, the immunosuppression management changed greatly (for example, from maintenance immunosuppression with cyclosporine before to now using tacrolimus) and this might have affected the rate of post-LT rejection rates.

In conclusion, the outcomes after ABO incompatible pediatric liver transplant have improved and are now comparable to ABO compatible transplants. ABO incompatible grafts should therefore be increasingly utilized by pediatric transplant programs to help increase the donor pool and reduce the waitlist mortality in children less than 12 months of age. Crossing blood groups in pediatric liver transplantation remains reserved for the sickest infants, which may affect post transplant outcomes. National policies need to be modified to allow small children to be eligible for an ABO incompatible transplant with a lower natural PELD score.

## Data Availability

The raw data supporting the conclusions of this article will be made available by the authors, without undue reservation.
